# Production of soluble mammalian proteins in Escherichia coli: identification of protein features that correlate with successful expression

**DOI:** 10.1186/1472-6750-4-32

**Published:** 2004-12-14

**Authors:** Michael R Dyson, S Paul Shadbolt, Karen J Vincent, Rajika L Perera, John McCafferty

**Affiliations:** 1 The Atlas of Gene Expression Project, The Wellcome Trust Sanger Institute, Wellcome Trust Genome Campus, Hinxton, Cambridge, CB10 1SA, UK

## Abstract

**Background:**

In the search for generic expression strategies for mammalian protein families several bacterial expression vectors were examined for their ability to promote high yields of soluble protein. Proteins studied included cell surface receptors (Ephrins and Eph receptors, CD44), kinases (EGFR-cytoplasmic domain, CDK2 and 4), proteases (MMP1, CASP2), signal transduction proteins (GRB2, RAF1, HRAS) and transcription factors (GATA2, Fli1, Trp53, Mdm2, JUN, FOS, MAD, MAX). Over 400 experiments were performed where expression of 30 full-length proteins and protein domains were evaluated with 6 different N-terminal and 8 C-terminal fusion partners. Expression of an additional set of 95 mammalian proteins was also performed to test the conclusions of this study.

**Results:**

Several protein features correlated with soluble protein expression yield including molecular weight and the number of contiguous hydrophobic residues and low complexity regions. There was no relationship between successful expression and protein pI, grand average of hydropathicity (GRAVY), or sub-cellular location. Only small globular cytoplasmic proteins with an average molecular weight of 23 kDa did not require a solubility enhancing tag for high level soluble expression. Thioredoxin (Trx) and maltose binding protein (MBP) were the best N-terminal protein fusions to promote soluble expression, but MBP was most effective as a C-terminal fusion. 63 of 95 mammalian proteins expressed at soluble levels of greater than 1 mg/l as N-terminal H10-MBP fusions and those that failed possessed, on average, a higher molecular weight and greater number of contiguous hydrophobic amino acids and low complexity regions.

**Conclusions:**

By analysis of the protein features identified here, this study will help predict which mammalian proteins and domains can be successfully expressed in *E. coli *as soluble product and also which are best targeted for a eukaryotic expression system. In some cases proteins may be truncated to minimise molecular weight and the numbers of contiguous hydrophobic amino acids and low complexity regions to aid soluble expression in *E. coli*.

## Background

The production of purified proteins is important for several experimental approaches aimed to assign gene function including antibody generation for immunocytochemistry and immunoprecipitation studies [1-3], *in vitro *mapping of protein – protein, protein – DNA or protein – RNA interactions [4,5] and structure determination [6]. The availability of proteins is also important for biomedical applications such as small molecule drug discovery and the production of therapeutic proteins and vaccines. In these situations it is essential to be able to reliably express the proteins in a heterologous system and purify them so that they possess the same folds and structure as they would in a natural *in vivo *state. To achieve this on a whole proteome scale a generic approach must be taken to the expression of protein families, unlike the traditional approach of protein chemistry in optimising the isolation of individual proteins on a case by case basis. *E. coli *has been the expression system of choice for the majority of laboratories engaged in high-throughput, multi-plexed cloning, expression and purification of proteins for structural genomics [7]. The advantages of *E. coli *as an expression host include well studied physiology, genetics and availability of advanced genetic tools [8-10], rapid growth, high-level protein production rates achieving up to 10–30% of total cellular protein, ease of handling in a standard molecular biology laboratory, low cost and the ability to multiplex both expression screening [11] and protein production [12]. There are however several disadvantages, particularly for eukaryotic proteins, of expression in a prokaryotic system. The lack of eukaryotic chaperones, specialised post-translational modifications, ability to be targeted to sub-cellular locations or to form complexes with stabilising binding partners can result in protein mis-folding and aggregation. For example, when 2078 randomly selected *C. elegans *full-length genes were cloned and expressed in *E. coli *only 11 % yielded soluble protein [13]. Similarly for 44 cloned human proteins, 12 were expressed solubly and 4 purified to homogeneity [14]. With the exception of full-length membrane proteins, the property of protein solubility has been shown to be a good indicator of correct folding as determined by functional binding [15,16] or enzymatic [17] assays. Purification of inclusion bodies and *in vitro *refolding has been used in a number of cases, but refolding conditions are highly protein specific and so unlikely to be useful for high-throughput protein expression.

There are several fall-back strategies for expression of correctly folded eukaryotic proteins in *E. coli *one of which is to truncate long multi-domain proteins into separate domains, as has been performed for the Ephb2 receptor [15,18,19]. Reducing translation rates so that proteins have an increased chance of folding into a native state prior to aggregating with folding intermediates, can be successful by lowering the temperature after induction [20] or inducing with lower concentrations of IPTG [21]. Alternate approaches include: co-expressing stabilising binding partners (see review [7]) or chaperones [22]; the induction of chaperones by heat shock [23] or chemical treatment [24]; or the use of genetically modified host-strains that can conduct oxidative protein folding in the cytoplasm [25,26], over-express rare tRNAs [27] or lipid rafts [28]. Perhaps one of the most successful generic strategies to enhance the expression of soluble proteins is the fusion with solubility enhancing tags, such as maltose binding protein (MBP), thioredoxin (Trx) and glutathione-S-transferase (GST) [29-31].

The aim of this work was to ask if it is possible to derive some general conclusions regarding which expression strategy would most likely result in the expression of soluble, functionally active mammalian protein on a family-by-family or domain-by-domain basis. A deep-mining approach was taken to maximise the chances of successful expression by examining the soluble expression of 30 different proteins using 14 different expression vectors. This study allowed us to make several conclusions regarding the best strategies to adopt for the soluble expression of different mammalian proteins in bacteria. The conclusions were tested by the expression of an additional 95 mammalian proteins.

## Results

### Expression clone construction

The 30 proteins chosen for this expression study are listed in Table 1. With the exception of GFP, they are all human or mouse proteins, and represent several diverse protein families with extra-cellular, cytoplasmic and nuclear cell locations. The list includes a mixture of full-length and truncated proteins expected to be easy or more challenging to express in a bacterial system. Protein truncations were designed to express individual domains annotated from the SwissProt [32] or Pfam [33] databases or following previous examples of successful expression [15]. The genes were isolated from cDNA using a nested PCR strategy [34] or provided by the FlexGene Consortium  and sequence confirmed. A recombinational cloning strategy was employed termed "GATEWAY" cloning [35,36] based on a modification of the phage lambda site-specific recombination system [37]. Primers were designed using the nearest neighbour algorithm [38] and open reading frames (ORFs) were PCR amplified from first strand cDNA with 5' attB1 and 3' attB2 linkers and then recombined with pDONR221 (Invitrogen) to give a set of entry clones which were sequence confirmed and then recombined with various destination vectors to give the expression constructs. Two sets of clones for each ORF were generated with and without stop codons for expression with N or C-terminal tags respectively. Recombinational cloning was useful in this study where the same set of ORFs could be cloned into a large set of different expression vectors without the requirement to check for compatible restriction sites in each vector or their absence within the ORFs.

**Figure 1:**
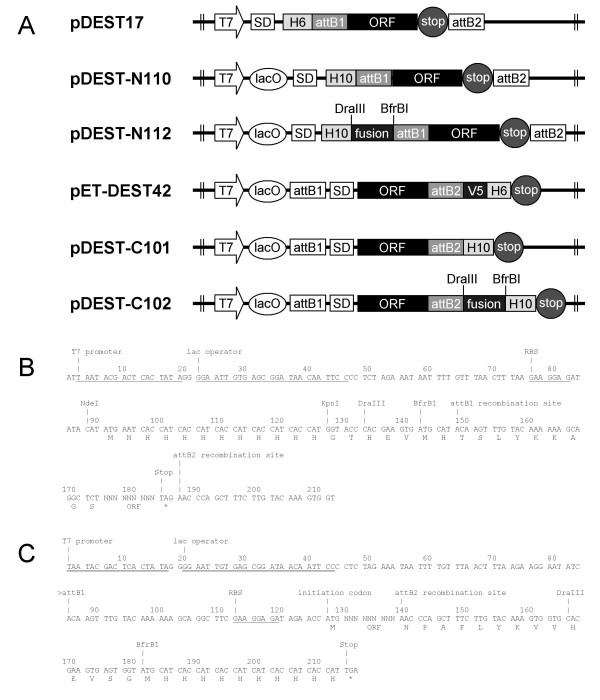
**Expression vector constructs after recombination between the destination and entry plasmids. **(A) Schematic representation where shaded and clear boxes indicate translated and untranslated regions respectively. T7 = T7 RNA polymerase promoter, lacO = lac operator, SD = shine dalgarno, H6 or H10 = hexahistidine or decahistidine, attB1 or attB2 = attB recombination sites, ORF = open reading frame, stop = stop codon, fusion = protein fusion (MBP, GFP, GST, Trx, DHFR or Dhfr), V5 = V5 epitope. (B) and (C) DNA sequences of pDEST-N112 and pDESTC102 respectively from T7 RNA polymerase promoter to stop codon.

For this study a set of destination vectors were constructed by modifying pET-DEST42 (see Materials and Methods). The T7 promoter was chosen over other promoters commonly used for bacterial expression because of the high specificity and processivity of T7 RNA polymerase and the wide choice of expression strains currently available. Briefly, multicloning sites were created either 5' of the attR1 or 3' of the attR2 recombination sites for insertion of DNA inserts encoding N or C-terminal tags respectively. The expression vectors contained a T7lac promoter [39] for improved control of basal expression. The N-terminal tag expression vectors contained a sequence at the translational start site to provide a partial match with the down-stream box (ATG AAT CAC CAT), shown to provide enhancement of translation [40] and a decahistidine (H10) tag for enhanced affinity for Nickel resins compared with hexahistidine (H6) tags (data not shown). A fusion partner was inserted between the H10 tag and recombination sites to examine the effect on soluble protein expression. Unlike previous tag comparisons [29-31] here the same promoter and 5'-UTR sequence was employed so that any expression differences observed would be purely due to the presence the fusion partner. A vector was also included in this study (pDEST17) with a T7 promoter and no downstream lac operator, which would add a H6 tag at the N-terminus (Figure 1).

### Effect of different N-terminal fusions on expression

**Figure 2:**
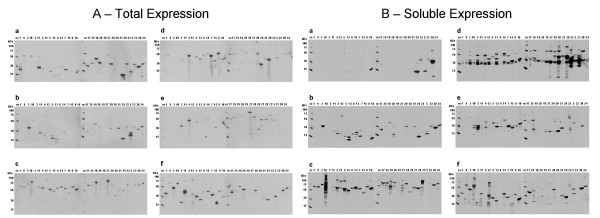
**Effect of N-terminal fusion on protein expression **Total (A) and soluble (B) expression for protein 1 – 30 (Table 1) with various N-terminal fusion partners analysed by SDS-PAGE fluorescence western blots as described in Materials and Methods. Expression plasmids employed were (a) pDEST17, (b) pDEST-N110 or pDEST-N112 with either (c) MBP, (d) GFP, (e) GST or (f) Trx inserted between the DraIII and BfrBI sites as shown in Figure 1.

Expression plasmids generated by recombination reactions were used to transform *E. coli *BL21(DE3), an expression strain containing chromosomally integrated T7 RNA polymerase gene (λDE3 lysogen) under the control of the lacUV5 promoter. To handle a large number of expression experiments (420 total) and associated manipulations to screen for total and soluble expression in E. coli, the recombinational cloning, transformation, growth of expression cultures and cell lysis and filtration separation of insoluble protein were performed in 96-well plate format. Figure 2 shows Western blots for total and soluble protein expression 2 hours after induction with 1 mM IPTG as described in Materials and Methods. The method for separating total from soluble proteins was based on that of Knaust and Nordlund [11] and consisted of detergent lysis of harvested cells followed by filtration through a 0.65 μm 96-well filter plate, which separates larger inclusion bodies from the soluble fraction. The filtration method agrees well with traditional centrifugation methods to separate soluble from insoluble protein [11,41] and has the advantage that multiple samples can be processed in parallel. Quantitation was achieved by separating the proteins by SDS-PAGE, electro-blotting onto PVDF membranes and detecting His tagged proteins with an anti-His5 monoclonal antibody followed by probing with an anti-mouse Cy-5 labelled antibody. The advantage of expression analysis by Western blot, compared to dot-blots, is that this allows one to quantitate the expression levels of full-length constructs and eliminate the contribution from cleaved protein tag. It was found that Western blots based on fluorescence detection [42] gave a greater dynamic range of detection compared with detection based on enzymatic amplification such as horse radish peroxidase (data not shown). A His-tagged protein molecular weight ladder was used for normalisation to eliminate any blot to blot variation. Table 2 shows the results of this analysis, quantitating expression yields in terms of mg expressed protein per litre of induction media for total and soluble expression. Expression yields greater than 2 mg/l are highlighted in bold.

Looking first at the results for total (soluble and insoluble) expression, no clear patterns emerge for the various expression vectors used. With the exception of CASP2, CDKN2A, Trp53, EGFR(TK), FOS and CD44 most proteins expressed well across all expression vectors. Interesting differences are apparent however when one looks at the production of soluble protein. Using decahistidine green fluorescent protein (H10-GFP) or decahistidine glutathione-S-transferase (H10-GST) as fusion partners at the N-terminus gave poor yields of soluble intact product. This may not be because they were poor at promoting soluble expression but because they were prone to proteolysis during cell lysis reducing the yield of full-length soluble protein. A set of proteins (GFP, RAF1(Ras-bd), HRAS, mdm2(p53-bd), Ephb2(TK) and CCND2) gave high soluble expression levels in the baseline N-terminal decahistidine vector, which was not improved when expressed as decahistidine thioredoxin (H10-Trx) or decahistidine maltose binding protein (H10-MBP) fusions. The molecular weight of these proteins ranged from 9 – 35 Kda and averaged 22.8 Kda. These proteins are all expressed in the cytoplasm, have an average of 1 low-complexity region, 3.8 contiguous hydrophobic amino acids (hp_aa), pI of 6.6, grand average of hydropathicity index (termed GRAVY[43] where increased positive number indicates increased hydrophobicity) of -0.32, 2.6% cysteine residues and no coiled-coil structures. A second grouping of proteins was observed where soluble expression was improved when expressed as H10-Trx or H10-MBP fusions compared with the H10 tag alone. This grouping included GRB2, Efnb2(EC1 or 2), MAD, MAX, Efna1 (FL and EC). The molecular weight of these proteins ranged from 16 – 25 Kda and averaged 20.5 Kda. These proteins were a mixture of those expressed in the cytoplasm, nucleus and extra-cellular, have an average of 0.71 low-complexity regions, 3.6 contiguous hydrophobic amino acids (hp_aa), pI of 6.8, GRAVY score of -0.79 and 1.7% cysteines. A third set of proteins resulted in almost undetectable soluble expression with a H10 tag but good expression with H10-Trx or H10-MBP fusions. These included CDK2, FLI1, CDKN-1B, mdm2, GATA2, Ephb2(LB) and CASP2 with molecular weights ranging from 22.5 – 54.5 Kda, with an average molecular weight of 40.4 Kda. These proteins were also a mixture cytoplasmic, nuclear and extra-cellular proteins, have an average of 2 low-complexity regions, 5 contiguous hydrophobic amino acids (hp_aa), pI of 6.9, GRAVY score of -0.55 and 2.3% cysteines. Finally a set of proteins was grouped (MMP1, FOS, EGFR(TK), Trp53, CD44) where very low (< 1 mg/l) soluble full-length expression was observed, even when expressed as MBP or Trx fusions. Here the molecular weight ranged from 40.7 – 81.6 Kda and averaged 51.4 kDa. These proteins were a mixture of those expressed in the cytoplasm, nucleus and extra-cellular, have an average of 3 low-complexity regions, 5.6 contiguous hydrophobic amino acids (hp_aa), pI of 5.7, GRAVY score of -0.50 and 1.8% cysteine content.

Comparing the 20 mammalian proteins where there are examples in all 6 expression vectors the average yields of soluble protein for the H10, H10-GFP, H10-GST, H10-Trx and H10-MBP tags are 3.3, 1.0, 1.4, 6.0 and 5.8 mg per litre of culture. This ranks the ability of the tag fusions to produce full-length soluble protein as H10-Trx ~ H10-MBP > H10 > H10-GST > H10-GFP. The pDEST17 vector (which encodes a H6 tag) was dramatically poorer at expressing soluble protein compared with the vector pN110 (which encodes a H10 tag), with average soluble expression yields of 0.8 and 3.3 mg per litre of culture respectively. Both vectors contain T7 RNA polymerase promoters, but pN110 also contains a lac operator (lacO) downstream of the promoter and the gene encoding the lac repressor (lacI) for tighter control of gene expression. This may result in a faster rate of transcript synthesis, after induction with IPTG, and hence translation rates (due to an increased concentration of mRNA) for pDEST17 compared with pN110. If translation rate exceeds the rate of protein folding, then increased production of insoluble protein would occur.

### Effect of different C-terminal fusions on expression

**Figure 3:**
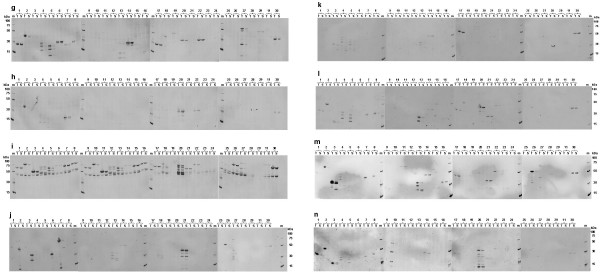
**Effect of C-terminal fusion on protein expression **Total (T) and soluble (S) expression for protein 1 – 30 (Table 1) with different C-terminal fusion partners analysed by SDS-PAGE fluorescence western blots as Figure 2. Expression plasmids employed were (g) pET-DEST42, (h) pDEST-C101 or pDEST-C102 with either (i) MBP, (j) GST, (k) GFP (l) Trx (m) Dhfr or (n) DHFR inserted between the DraIII and BfrBI sites as shown in Figure 1.

A similar study was performed where the 30 ORFs were cloned into 8 different C-terminal tag expression vectors shown in Figure 1. C-terminal fusions studied here included V5-H6 or H10 or protein fusions MBP, GST, Trx, murine or human dihydrofolate reductase (Dhfr or DHFR respectively), all with H10 at the C-terminus. The expression screen and quantitation of total and soluble protein expression was performed as for the N-terminal tag study. Figure 3 shows the fluorescence western blots for this C-terminal tag study. Here a greater number of constructs were observed with either undetectable or low levels of expression compared with the N-terminal tag study. Table 3 quantitates the Western blot data for the intact fusion products, with expression yields greater than 2 mg/l in bold. The last row of the table describes the average expression yield for each C-terminal fusion partner. For total protein expression levels there are large expression level differences observed between the various C-terminal tags. The C-terminal decahistidine tag was particularly poor here with an average total expression yield of only 0.7 mg/l compared with 7.3 mg/l when this tag was fused to the N-terminus. In contrast the C-terminal MBP-H10 tag resulted in an average total expression yield of 20.2 mg/l. The ranking of the C-terminal fusion partners in promoting total expression was MBP-H10 > GST-H10 > V5-H6 > Trx-H10 > Dhfr-H10 > DHFR-H10 > GFP-H10 > H10.

MBP-H10 was the most effective tag at the C-terminus to promote protein solubility with an average construct full-length soluble yield of 5.0 mg/l, which compares well with an average of 5.8 mg/l when this tag is fused at the N-terminus. The order of C-terminal tags to promote soluble expression was similar for total expression: MBP-H10 > GST-H10 > V5-H6 > Dhfr-H10 ~ GFP-H10 ~ Trx-H10 > H10 ~ DHFR-H10. Thioredoxin was not as effective a solubility enhancing tag when fused at the C-terminus with an average soluble yield of only 0.7 mg/l compared with 6.0 mg/l when fused to the N-terminus.

Several correlations with protein features are seen when one groups the MPB fusions according to soluble protein expression levels. For the first group, where soluble expression levels were in the range of 5 – 50 mg/l, the average molecular weight, pI and GRAVY score were 20.6 KDa, 5.9 and -0.58 respectively. The average numbers of contiguous hydrophobic amino acids, low complexity and coiled-coil regions were 3.1, 0.56 and 0.22 respectively. The second group displayed soluble expression levels between 1 – 5 mg/l. Here, the average molecular weight, pI and GRAVY score were 25.1 KDa, 7.9 and -0.39 respectively and the average numbers of contiguous hydrophobic amino acids, low complexity and coiled-coil regions were 4.3, 0.71 and 0 respectively. The last group displayed soluble expression levels between 0 – 1 mg / l. Here the average molecular weight, pI and GRAVY score were 41.1 KDa, 6.2 and -0.51 respectively and the average numbers of contiguous hydrophobic amino acids, low complexity and coiled-coil regions were 5, 2.43 and 0.21 respectively. There were representatives of nuclear, cytoplasmic and extra-cellular proteins in all three groupings.

### Expression of a test set of 95 mammalian proteins

A diverse set of proteins were chosen to test the conclusions of this study (Table 4). They range from proteins that are well annotated, some of which have been expressed in *E. coli *previously (Nfkb1), to those that contain no PfamA domains and have not been expressed in *E. coli *previously (Maat1, BC031407, Ttyhl, 1500001H12RIKEXT2, Ext2, KIAA1136, G2 and KIAA1549). They included 24 proteins not annotated as PfamA domains, with unknown function. All cDNAs were amplified from a primary cDNA library, cloned into pDONR221 and sequence confirmed prior to transfer to pDEST-N112-MBP (Figure 1) for expression as N-terminal H10-MBP fusions. In some cases primers were designed to clone protein fragments to express particular PfamA domains or minimise the molecular weight or numbers of low complexity (LC) regions or contiguous hydrophobic amino acids (hp_aa). For proteins with no PfamA annotations, such as BC031407, SMART sequence analysis [44] was performed to identify the low complexity regions of the protein and truncations performed accordingly. Protein expression and quantitation of intact soluble fusion protein product was performed as for the N- and C-terminal tag comparison study. The total and soluble expression levels (mg of protein per litre culture) are listed in the last column of Table 4 together with selected protein features. 63 of the 95 proteins yielded soluble expression levels of greater than 1 mg/l and the average molecular weight, number of LC regions and hp_aa for these proteins was 24.4 kDa, 0.9 and 3.7 respectively. For the 32 proteins that failed to give soluble product of the correct size, the average molecular weight, number of LC regions and hp_aa was 37.1 kDa, 1.8 and 4.5 respectively.

## Discussion

### Correlation between protein properties and solubility

To guide future expression strategies for new proteins, particularly regarding the choice of expressing a full-length protein in a bacterial or eukaryotic system and also where to truncate multi-domain containing proteins, it is interesting to investigate if the proteins expressed in a soluble form in this study share any common properties. Recently Goh *et al. *[45] used data generated by a structural genomics consortium to examine the ability of proteins to progress from cloning to expression and purification to crystallisation. The data used was very large, consisting of 27,000 targets from over 120 organisms and a number of important features were inferred that correlated with success including percentage composition of charged residues, occurrence of hydrophobic patches and length. Although a large study, there was a problem with interpretation of all the data-sets as it was unclear whether targets were simply waiting in the pipeline or had failed. Also structural genomics targets are often initially biased in favour of easy to express proteins, not representative of the whole proteomes of these organisms.

The present study, focused on mammalian proteins from several diverse families, examined the relationship between successful soluble expression with various protein properties. Several protein features were identified in this study to correlate with soluble expression, which had not previously been shown experimentally. For both the N and C-terminal tag expression studies it was observed that the presence of several features did not correlate with successful expression including protein pI, grand average of hydropathicity index (GRAVY) [43], sub-cellular location, the cysteine content as a percentage of the total number of amino acids and the number of coiled-coils. Protein pI has been linked to sub-cellular location [46] with a bimodal distribution observed in bacterial and archaeal genomes and trimodal pattern in eukaryotes. Proteins are thought to be less soluble at a pH environment near their pI. GRAVY simply calculates overall hydrophobicity of the linear polypeptide sequence with increasing positive score indicating greater hydrophobicity, but no account is taken of the way the protein folds in three dimensions or the percentage of residues buried in the hydrophobic core of the protein. In a recent study Luan *et al. *[47] tested the soluble expression of 10,167 full-length *C. elegans *ORFs and found that protein hydrophobicity was an important factor for an ORF to yield a soluble expression product. This different result may be attributable to the fact that the *C. elegans *study included a greater proportion of membrane proteins. Therefore the lack of correlation between GRAVY score and soluble expression we observed may be true for non-membrane proteins or for proteins where the trans-membrane domain has been deleted.

There was a strong correlation between successful soluble expression and molecular weight of the protein. Small proteins with an average molecular weight of 22.8 KDa did not require to be fused with solubility enhancing proteins for soluble expression whereas proteins that required to be fused with N-terminal MBP or Trx for soluble expression had an average molecular weight of 40.4 KDa and those where the addition of a N-terminal fusion could not rescue soluble expression had an average size of 51.4 KDa. The same pattern also emerged in the C-terminal fusion study. The decreasing probability of successful soluble expression of mammalian proteins with increasing molecular weight is likely due to increasing protein complexity, perhaps requiring specialised eukaryotic chaperones for folding or stabilising binding partners. The majority of proteins solubly expressed in this study contained single domains and as fusion proteins were either capable of self-folding or were folded with the aid of prokaryotic chaperones. Braun *et al. *found a similar relationship with their set of 32 human proteins with 4 different N-terminal fusions [30].

A correlation in this study was observed between increasing numbers of contiguous hydrophobic amino (hp_aa) acids (AILFWV) and soluble expression. This ranged from an average of 3.8 hp_aa for those proteins not requiring a N-terminal fusion for high level soluble expression to 5 hp_aa for proteins requiring a N-terminal fusion for successful expression and 5.6 hp_aa where expression failed under the conditions described here. This pattern was also repeated in the C-terminal fusion study where good expression proteins had an average of 3.1 hp_aa whereas poor expression proteins had an average of 5 hp_aa. In a study of the sequences of 2753 non-membrane proteins it was found that the sequences of three or more consecutive hydrophobic residues are suppressed in globular proteins [48]. Low complexity regions of proteins are regions of a protein of biased composition containing a small number of amino acids [33] and can have a disordered structure important for protein function [49]. Here we found that the greater the number of low complexity regions contained within the target protein, the less likely soluble expression would be achieved. This was true for both the N- and C-terminal fusion protein studies with 0.6 – 1 low complexity regions for proteins easy to express in a soluble form to 2.4 – 3 low complexity regions for proteins difficult to express. Low complexity regions are less common in bacterial proteins and these may be targets for proteolytic degradation *in vivo*.

Some interesting conclusions were drawn when soluble expression was measured for an additional set of 95 mammalian proteins expressed as H10-MBP fusions (Table 4). In several cases (ELF1, Fli1, Ldb1, BC031407, Nfkb1 and RelA-p65) truncating the proteins to minimise the molecular weight and the numbers of low complexity regions and contiguous hydrophobic amino acids made the difference between failed expression and good soluble protein expression. For proteins such as BC031407, with no annotated PfamA domains, it was found that truncating at low complexity regions was a good method to identify a fragment that could express in a soluble form of the correct size (protein 81). Although we found that successful soluble expression of the 95 protein set correlated with lower molecular weight, number of low complexity regions and contiguous hydrophobic amino acids compared with proteins that failed to express solubly with the correct size, validating our earlier conclusions, there were some exceptions. For example Elf1 and Gata1 both expressed well despite having 4 and 6 low complexity regions respectively and molecular weights of 66 and 42.5 kDa, whereas some smaller proteins such as the PDZ domains of Dlgh3 and Grip1 failed to express. It may be that there are additional protein features, such as the ability to form a stabilising interaction with a binding partner, that are also important for soluble expression. Also ensuring correct protein domain boundaries may be important since the annotated Pfam domain boundaries, based on sequence alignment, do not always match the structural or folding domain boundaries.

### Protein fusions that enhance protein solubility

There have been three comparative studies recently where sets of proteins were cloned into several expression vectors and the effects of the fusion partner on total and soluble expression yield were examined. Hammarstrom *et al. *[29] cloned 27 human proteins (MW < 20 Kda) into various expression vectors and ranked the tags ability to promote soluble expression as Trx ~ MBP ~ Gb1 > ZZ > NusA > GST > His6. Another study ranked tags in terms of increased expression and yield after purification as GST ~ MBP > CBP > His6 when comparing the expression of 32 human proteins where the molecular weight varied from 17 – 110 kDa.[30] Here GST was preferred because of the weak affinity between MBP and amylose resin. In a third study of 40 different proteins (10 mammalian, 3 plant and 2 insect) with 8 different tags MBP gave the best overall results in terms of total and soluble expression [31]. However, these studies used different combinations of promoter and fusion partner, so it was unclear whether the observed effect was purely due to expression with the fusion partner or variable rates of transcript synthesis that would also affect translation rates.

In this study it was found that, on average, N-terminal fusion partners are preferable for optimal protein expression. When proteins are expressed with their native N-terminus, as in our C-terminal fusion proteins, total expression levels can be more variable than when expressed with a constant N-terminal tag. This may be because of variable RNA secondary structures in the region around the start codon which could interfere with ribosome binding. An additional explanation is that during translation the expressed protein emerges from the ribosome first and initiates an incorrect, irreversible, folding pathway before the soluble fusion partner has been translated and folded. The mis-folded protein would be ubiquitin labelled and targeted to the proteasome for degradation resulting in lower total expression levels. This scenario is more likely when expressing mammalian proteins in a bacterial system which lacks specific eukaryotic chaperone proteins. It has been shown previously that proteins prone to mis-folding and aggregation can arrest GFP folding when fused at the C-terminus [17]. However, when the soluble protein is fused at the N-terminus, this would be translated first and perhaps increase the solubility of the downstream protein domain folding intermediates, increasing their half lives prior to irreversible aggregation. This would allow greater reversibility in the individual steps along the folding pathway and increase the probability that the protein would eventually reach the lowest free energy native conformation.

It was found that Trx and MBP were the best N-terminal protein fusions to promote protein solubility. The best C-terminal fusion to promote protein solubility was MBP and this may be acting as a true intra-molecular chaperone [50], able to promote folding of the N-terminal protein fusion. The mechanism could be due to direct binding to folding intermediates [51], allowing stabilisation prior to correct folding and inhibition of aggregate formation. The observation that MBP was effective at enhancing soluble expression when fused at the C-terminus, in contrast to thioredoxin, suggests that MBP can actually reverse the process of incorrect folding that would have started prior to the translation of the downstream MBP. This property was not observed for thioredoxin when fused to the C-terminus suggesting either that, in three-dimensions, different proximal faces of the fusion partners have different solubility enhancing properties or that thioredoxin does not posses any chaperone properties and acts only as a solubility enhancer. Alternatively, the folding of thioredoxin may be more prone to inhibition than MBP. Also there are examples where MBP fusions can form soluble inclusion bodies [52,53], and this cannot be ruled out as a possibility here, although there are also several examples where MBP fusion proteins are fully functionally active [50,52,54,55].

It must be stressed here that although protein solubility is a useful indicator of correct folding, additional measurements need to be performed to give supporting evidence for correct folding. These may include removing the protein fusion with a protease and analysis of the cleaved protein of interest by a variety of biophysical and functional assays such as analysis of monodispersity by light scattering [52], NMR [56,57], CD spectropolarimetry, bis-ANS binding [53], ligand binding or enzymatic activity. In this study a protease cleavage site was not included in the vector constructs because the main use of the proteins generated in our laboratory will be in high-throughput antibody production where the cleavage of the fusion partner is unnecessary.

GFP did not significantly enhance soluble protein expression when fused to the C-terminus of the proteins in this study, supporting the use of this tag as an indicator of soluble protein expression of fused ORFs.[17,41] The observation that the V5-His6 tag resulted in a higher average soluble expression level than the His10 tag (1.7 compared with 0.3 mg/l) indicates that the identity of the peptide tag can also affect overall solubility of expressed proteins.

## Conclusions

What guidelines have emerged from this study in developing a strategy for the production of soluble mammalian proteins in *E. coli*? If the protein has a molecular weight of less than 30 KDa and contains 1 or less low complexity regions and less than 4 contiguous hydrophobic amino acids expression of the full-length protein in *E. coli *should give good levels of soluble protein. As a generic strategy we would recommend expressing the protein with a fusion partner and found MBP and Trx to be the best fusions to enhance protein solubility as N-terminal tags with MBP being superior as a C-terminal fusion. C-terminal fusions are desirable for proteins such as the P450s where N-terminal tags can inhibit functional activity. When fused to an optimal fusion partner, nuclear, cytoplasmic and extra-cellular domains were equally likely to be expressed solubly. For larger proteins over 50 KDa, truncations should be considered to express specific protein domains and to minimise the molecular weight, number of low complexity regions and contiguous hydrophobic amino acids. In conclusion, this study will help enable a systematic expansion in the number mammalian proteins and domains that can be successfully expressed in *E. coli *as soluble product, and also predict which are best targeted for a eukaryotic expression system.

## Methods

### Materials

Oligonucleotides were synthesised by Qiagen-Operon (Cologne, Germany) or Sigma-Genosys (Haverhill, UK). All restriction enzymes were from New England Biolabs (Hitchin, UK). The vectors pET-DEST42, pDEST17 and pDONR201 and *E. coli *DB3.1 and BL21(DE3)Star pLysS, Gateway BP and LR clonase enzyme mix, pre-cast 4–12 % NuPAGE Bis-Tris gels and PVDF membranes (0.45 μm pore size) were all from Invitrogen (Paisley, UK). Entry plasmids in both open (minus stop codon) or closed format (plus stop codon) containing the full-length genes for GRB2, HRAS, JUN, FOS, MAD, MAX, CDK2, CDK4, CDKN1B, CASP2, MMP1, CDKN2A and CD44 were provided by Pascal Braun and Josh LaBaer (Harvard Institute of Proteomics, Cambridge, USA). A full length clone containing the full-length human EGFR ORF was provided by the RIKEN BioResource Center (Tsukuba, Japan) and Efna1 from the Mammalian Gene Collection (MGC) archived at the Wellcome Trust Sanger Institute (Hinxton, UK). First strand synthesis human and mouse cDNA was from BD Biosciences (Oxford, UK). Plasmid, gel extraction and PCR purification kits and 6xHis protein ladder were purchased from Qiagen (Crawley, UK). The expression strain BL21(DE3), BugBuster protein extraction reagent and His tag monoclonal antibody was from Merck Biosciences (Nottingham, UK). The 96-well multiscreen-DV durapore filter plate with 0.65 μm pore size was from Millipore (Watford, UK) and Cy5-labelled goat anti-mouse IgG from Amersham Biosciences (Little Chalfont, UK). Europium labelled antibodies and DELFIA reagents were from Perkin Elmer (Beaconsfield, UK) and all other chemicals unless otherwise stated were from Sigma-Aldrich (Gillingham, UK).

### N-Terminal fusion GATEWAY destination vector construction

To prepare pET-DEST42-MCS, a multi-cloning site was inserted into pET-DEST42 (Invitrogen) at nt396, between the shine-dalgarno sequence and the attR1 recombination site, encoding the recognition sequences for NdeI, KpnI, DraIII and BfrBI. Inverse or whole plasmid PCR was performed on pET-DEST42 with 5'-phosphorylated PAGE purified primer pairs 20 (5' TACCCACGAAGTGATGCATACAAGTTTGTACAAAAAAGCTGAACG 3') and 21 (5' CCCATATGTATATCTCCTTCTTAAAGTTAAACAAAATTATTTCTAGAG 3') in a 20 μl reaction containing 10 ng pET-DEST42, 0.3 μM primers 20 and 21, 20 mM Tris-HCl (pH 7.5), 0.5 mM DTT, 200 μM each of dATP, dCTP, dGTP and dTTP, 1 mM MgSO_4_, and 0.5 unit KOD hot start DNA polymerase (Novagen). PCR cycling conditions were: 94°C – 2 mins followed by 15 cycles of 94°C – 15 s, 59°C – 30 s, 68°C – 9 mins. The 7468 bp PCR product was purified using a PCR purification spin column (Qiagen) and eluted with 30 μl of 10 mM Tris-HCl (pH8.5), digested with 20 units of DpnI enzyme at 37°C for 4 hrs, to remove methylated plasmid DNA, purified by spin column and an intramolecular ligation reaction performed using 16 ng of linear PCR product and 5 units T4 DNA ligase and the buffers from the rapid ligation kit (Roche). The ligated PCR product was used to transform *E. coli *DB3.1 and the resultant pET-DEST42-MCS plasmid DNA prepared and sequence confirmed. Insert 1, encoding a decahistidine tag with a 5'-NdeI site and blunt 3' end, was prepared by PCR with primer pairs 22 (5' GGAATTCCATATGAAUCAC 3') and 24 (5' pGTGATGGTGATGGTGATGGTGATGGTGATTCATATGGAATTCC) and insert 2 encoding a decahistidine tag flanked by a 5'-NdeI site and 3'-KpnI site was prepared with primer pairs 22 and 26 (5' CGGGGTACCATGGTGATGGTGATGGTGATGGTGATGGTGATTCATATGGAATTCC 3'). PCR reactions were as above except the annealing temperature dropped to 44°C, extension time to 10 s and 12 cycles employed. Insert size was checked by 10 % TBE-PAGE and purified by a nucleotide removal kit (Qiagen). Expression vectors (b) pDEST-N110 and pDEST-N112 (Figure 1) were prepared by digestion of inserts 1 and 2 with NdeI only or NdeI and KpnI combined respectively, purified by spin column and ligated in a 1:1 ratio to NdeI, BfrBI or NdeI, KpnI digested pET-DEST42-MCS respectively prior to transformation of *E. coli *DB3.1. Inserts encoding MBP, GFP, GST or Trx flanked by a 5' DraIII site and a 3' blunt end were generated by PCR amplification from the plasmids pMALc2 (New England Biolabs), pET41a or pET32 (Novagen) respectively The primer pairs for MBP were 78 (5' TTATTACACGAAGTGAAAATCGAAGAAGGTAAACTGGTAATC 3') and 79 (5' pGTTCGAGCTCGAATTAGTCTGCGCGTCTTTC), for GFP 84 (5' TTATTACACGAAGTGGCTAGCAAAGGAGAAGAACTTTTCACTGGAG 3') and 85 (5' pTTTGTAGAGCTCATCCATGCCATGTGTAATC 3'), for GST 86 (5' TTATTACACGAAGTGTCCCCTATACTAGGTTATTGGAAAATTAAGGG 3') and 87 (5' pATCCGATTTTGGAGGATGGTCGCCACC 3') and for Trx 88 (5' TTATTACACGAAGTGAGCGATAAAATTATTCACCTGACTGAC 3') and 89 (5' p CAGGTTAGCGTCGAGGAACTCTTTC 3'). The inserts were digested with DraIII and ligated with DraIII, BfrBI cut pDEST-N112 vector to create the GATEWAY destination vectors pDEST-112-MBP, pDEST-112-GFP, pDEST-112-GST, pDEST-112-Trx.

### C-Terminal fusion GATEWAY destination vector construction

pDEST-C101 was designed to insert a decahistidine encoded sequence between the attR2 recombination site and T7 transcription termination region. pDEST-C102 is as C101 except a DraIII, BfrBI site was inserted downstream of the attR2 recombination site. Inverse PCR was performed as described above with primer pairs 1 (5' pCACCATCACCATCATCACCATCACCATTGAGTTTGATCCGGC) and 2 (5' pATGCACCACTTTGTACAAGAAAGCTGAAC) to generate pDEST-C101 and primer pairs 1 and 3 (5' pATGCATACCACTCACTTCGTGCACCACTTTGTACAAGAAAGCTGAAC) to prepare pDEST-C102. Murine and human dihydrofolate reductase (Dhfr and DHFR respectively) inserts flanked by a 5' DraIII site and blunt end at the 3' were amplified from MGC clones using the primer pairs 82 (5' TTATTACACGAAGTGCGACCATTGAACTGCATCGTCGCCGTG) and 83 (5' pGTCTTTCTTCTCGTAGACTTCAAACTTATAC 3') for Dhfr and 80 (5' TTATTACACGAAGTGGGTTCGCTAAACTGCATCGTCGCTGTG) and 81 (5' pATCATTCTTCTCATATACTTCAAATTTG) for DHFR. The DraIII digested inserts were ligated with DraIII, BfrBI digested pDEST-C102 vector to create pDEST-C102-MBP, GFP, GST, Trx, Dhfr and DHFR as shown in Figure 1.

### cDNA isolation and expression clone generation

A nested PCR strategy was used to isolate protein encoding ORFs directly from cDNA adapted for GATEWAY cloning from the method described by J. E. Collins *et al. *[34]. Briefly 2 sets of primer pairs were designed, the first pair of optimised primers binding 1 – 200 bp 5' and 3' of the ORF using DS-Gene software (Accelerys) and a second set of primers targeted to the beginning and end of the ORF. All primers were designed with melting temperatures around 60°C. PCR 1 contained 50 pg of either human universal QUICK-clone II cDNA (Clontech) or 50 pg of a mixture of mouse brain, heart, kidney, liver, smooth muscle, spleen, testis and 7, 11, 15 and 17-day embryo QUICK-clone cDNA (Clontech), 0.25 μM primers, 20 mM Tris-HCl (pH 7.5), 0.5 mM DTT, 200 μM each of dATP, dCTP, dGTP and dTTP, 1 mM MgSO_4_, and 0.5 unit KOD hot start DNA polymerase (Novagen) in a total volume of 20 μl. The PCR reaction consisted of 94°C – 2 mins, and 30 cycles of 94°C – 15 s, 55°C – 30 s, 68°C – 2.5 mins followed by 68°C – 5 mins. A 50-fold dilution of the PCR 1 reaction was made for the second 30 cycle PCR containing the ORF specific primers. Linkers were added to these primers encoding half the attB1 and attB2 sites for forward and reverse primers respectively. For entry clone generation to be transferred to N-terminal tag expression vectors the 5'-linkers for the forward and reverse primers were 5' AAAAAGCAGGCTCT 3' and 5' AGAAAGCTGGGTTCTA 3' respectively with the reverse primer adding a stop codon. For inserts destined to the C-terminal tag expression vectors the forward and reverse primers were 5' AAAAAGCAGGCTTCGAAGGAGATAGAACCATGG 3' and 5' AGAAAGCTGGGTT 3' respectively with the forward primer encoding the shine-dalgarno and kozak sequences and start codon. PCR 2 products were analysed by 1 % TBE-agarose electrophoresis[58] and correct size fragments were then subjected to an adapter PCR step to complete the flanking attB1 and attB2 sites. This consisted of a PCR reaction as described above using 1 μl of a 50-fold dilution of the PCR 2 reaction in a total volume of 20 μl and primer pair 113 (5' GGGGACAAGTTTGTACAAAAAAGCAGGCT 3') and 114 (5' GGGGACCACTTTGTACAAGAAAGCTGGGT 3') except that the annealing temperature was 45°C, only 12 cycles were used and extension time was 2 mins. The products of the adapter PCR were purified by a 96-well PCR clean-up kit (Qiagen), eluted in 100 μl 10 mM Tris-HCl (pH8.5) and had an average concentration of 40 ng /μl. Recombinational cloning of attB flanked PCR products with an attP containing pDONR vector to generate a set of entry plasmids was as described previously [35] except that pDONR221 (Invitrogen) was used. The ORFs within sequence confirmed attL containing entry plasmids were then recombined the various attR destination vectors described above to generate sets of expression plasmids. The LR recombination reactions [35] were used to transform E. coli DH5α cells, miniprep plasmid DNA prepared and this used to transform the various BL21(DE3) expression strains used in this study.

### Expression screening and quantitation

All BL21(DE3) transformants were selected and propagated in the presence of 100 μg/ml ampicillin. A single antibiotic resistant colony was used to inoculate 0.5 ml 2xYT media in a 96-deep well block containing the appropriate antibiotics and shaken at 210 rpm at 37°C. When the average OD_600 _had reached 1 (3 hrs for BL21(DE3)), 60 μl was transferred to 1.2 ml 2xYT media in a 96-deep-well block containing the appropriate antibiotics, placed on a shaking incubator at 37°C and when the OD_600 _reached 0.5 (2 hrs for BL21(DE3)) IPTG added to a final concentration of 1 mM and shaking continued at 25°C for 12 hours. Total protein was analysed by transferring a 20 μl aliquot of the induced culture to a 96-well PCR plate containing 20 μl of 2 × NuPage LDS loading buffer (Invitrogen), 0.1 M DTT, heated to 95°C for 10 mins and cooled on ice prior to loading 10 μl on a 17-well 4–12 % NuPAGE Bis-Tris gels with a multi-channel gel loading syringe (Hamilton). Soluble protein was extracted by transferring 290 μl of induced culture to a shallow well plate, centrifugation at 3000 g for 5 mins, supernatant removed and cells were resuspended in 58 μl BugBuster containing 1.4 units of benzonase and 58 units of recombinant lysozyme (Novagen). For the C-terminal tag and expression strain comparison this buffer was also supplemented with 0.58 μl protease inhibitor cocktail set III 10-fold diluted in DMSO (Novagen). The cell-pellets were resuspended with a multi-channel pipette and incubated with slow shaking for 20 mins at room temperature prior to transfer to 96-well multiscreen-DV durapore filter plates with 0.65 μm pore size (Millipore). The filter plate was placed on top of a shallow 96-well plate and centrifuged at 1000 g for 2 mins. 4 μl of the filtrate was then added to a 96-well plate containing 5 μl of 4 × NuPage LDS loading buffer (Invitrogen), 11 μl of 182 mM DTT, the plate heated at 95°C for 5 mins and loaded onto a 17-well 4–12 % NuPAGE Bis-Tris gel. A His-tagged molecular weight ladder (Qiagen) was also loaded onto each gel. Gel electrophoresis and electro-transfer to PVDF membrane was as described.[58] Blots were blocked with 3 % Marvel milk powder in PBS-Tween (PBS with 0.1% Tween) either 1 hour at room temperature or over-night at room-temperature, washed with PBS-Tween and incubated with 40 ng/ml anti-His5 tag monoclonal antibody (Novagen), 3 % Marvel, PBS-Tween for 1 hr, washed 3 × PBS-Tween, incubated with 1 μg/ml Cy5 labelled goat anti-mouse in 3% Marvel, PBS-Tween for 1 hr, washed 3 × PBS-Tween and 2 × PBS and blots dried at 37°C for 10 mins between blotting paper. The blots were scanned on a Typhoon 8600 variable mode imager (Amersham) with fluorescence scan mode, 633 nm excitation laser, 670 nm emission filter, 600 V PMT and 200 μm / pixel scan resolution. The integrated fluorescence intensity volumes of bands on the gel were quantitated using ImageQuant TL software (Amersham). Conversions to protein yield were made by using a calibration curve of purified His-tagged single chain antibody (scFv). Differences between the molecular weight (MW) of the scFv (31 KDa) and each expressed fusion protein were taken into account by multiplying each protein quantitation by the ratio MW construct (KDa) / 31. The numbers were normalised to eliminate blot to blot variation using a His-tagged molecular weight ladder (Qiagen).

## Authors' contributions

MRD performed the molecular biology, participated in the bioinformatics, expression screening, quantitation, experimental design and drafted the manuscript. SPS and RLP participated in the expression screening and quantitation. KJV helped with the bioinformatics (database searching, protein domain annotation and primer design). JM participated in the experimental design, coordination and helped to draft the manuscript. All authors approved the final manuscript.

## Figures and Tables

**Table 1:** Proteins for expression study with selected features

No	Protein^a^	Domain^b^	Construct^c^	Organism^d^	Protein Family^e^	MW (Kda)	pI	Cys %	GRAVY^f^	hp_aa^g^	Sub-cellular Location	LC^h^	CC^i^
26	CASP2	FL	1–435/435	Hs	CARD, Peptidase_C14	48.9	6.3	4.1	-0.30	5	Cytoplasm	1	0
24	CCND2	FL	1–289/289	Hs	cyclin, cyclin_C	33.1	4.9	4.1	-0.21	4	Cytoplasm	2	0
29	CD44	FL	1–742/742	Hs	Xlink, Pfam-B × 9	81.6	5.0	1.2	-0.77	10	Extra-cellular	9	0
22	CDK2	FL	1–298/298	Hs	pkinase	33.9	8.9	1	-0.08	4	Cytoplasm	0	0
23	CDK4	FL	1–303/303	Hs	pkinase	33.7	6.6	1.3	-0.17	4	Cytoplasm	0	0
25	CDKN1B	FL	1–198/198	Hs	CDI, Pfam-B × 2	22.1	6.6	2	-1.26	2	Cytoplasm	0	1
28	CDKN2A	FL	1–156/156	Hs	ank	16.5	5.4	0.6	-0.23	4	Cytoplasm	0	0
6	Efna1	FL	18–205/205	Mm	Ephrin	21.9	6.4	2.1	-0.59	8	Extra-cellular	1	0
7	Efna1	EC	18–154/205	Mm	Ephrin	16.2	6.5	2.9	-0.86	2	Extra-cellular	0	0
5	Efnb2	EC1	29–176/336	Mm	Ephrin	16.6	5.3	2.7	-0.47	3	Extra-cellular	0	0
4	Efnb2	EC2	29–210/336	Mm	Ephrin	20.1	8.6	2.2	-0.64	3	Extra-cellular	0	0
15	EGFR	TK	694–1022/1210	Hs	Pkinase, Pfam-B	37.3	5.5	1.8	-0.22	3	Cytoplasm	1	0
8	Epha2	LB	24–206/977	Mm	EPH_lbd	21.1	4.7	2.7	-0.30	4	Extra-cellular	0	0
1	Ephb2	LB	28–210/994	Mm	EPH_lbd	22.5	5.8	2.2	-0.14	4	Extra-cellular	0	0
3	Ephb2	SAM	922–994/994	Mm	SAM_1	8.3	4.9	0	-0.03	2	Cytoplasm	0	0
2	Ephb2	TK	595–906/994	Mm	Pkinase	35.3	5.6	1.6	-0.27	5	Cytoplasm	0	0
10	Fli1	FL	1–452/452	Mm	Ets, SAM_PNT, Pfam-B × 5	51.0	6.6	0.9	-0.79	3	Nuclear	1	0
19	FOS	FL	1–380/380	Hs	bZIP, Pfam-B × 4	40.7	4.6	2.1	-0.37	5	Nuclear	5	1
9	GATA2	FL	1–480/480	Hs	GATA	50.3	9.7	2.7	-0.51	13	Nuclear	7	0
30	GFP	FL	1–238/238	Av	GFP	26.9	5.6	0.8	-0.52	3	Cytoplasm	0	0
14	GRB2	FL	1–217/217	Hs	SH2, SH3	25.2	5.9	0.9	-0.67	5	Cytoplasm	0	0
17	HRAS	FL	1–189/189	Hs	ras	21.3	5.0	3.2	-0.42	4	Cytoplasm	1	0
18	JUN	FL	1–331/331	Hs	bZIP, Jun	35.7	9.0	0.9	-0.47	3	Nuclear	3	1
20	MAD	FL	1–221/221	Hs	HLH, Pfam-B × 2	25.3	8.9	1.4	-0.97	2	Nuclear	3	1
21	MAX	FL	1–160/160	Hs	HLH, Pfam-B × 2	18.3	5.9	0	-1.32	2	Nuclear	1	1
12	Mdm2	FL	1–489/489	Mm	SWIB, zf-RanBP, Pfam-B × 8	54.5	4.5	3.5	-0.83	4	Nuclear / Cytoplasm	5	0
13	Mdm2	p53-bd	19–230/489	Mm	SWIB, Pfam-B × 2	11.7	8.8	0.5	-0.25	4	Nuclear / Cytoplasm	3	0
27	MMP1	FL	1–469/469	Hs	Peptidase_M10_N, Peptidase_M10, Hemopexin	54.0	6.5	0.6	-0.57	7	Extra-cellular	0	0
16	RAF1	Ras-bd	51–131/648	Hs	RBD	9.2	9.9	3.8	-0.30	3	Cytoplasm	0	0
11	Trp53	FL	1–390/390	Mm	P53	43.5	7.0	3.1	-0.59	3	Nuclear / Cytoplasm	1	0

^a^LocusLink symbol. ^b^Domain: LB, ligand binding; TK, tyrosine kinase; SAM, sterile alpha motif; EC, extra-cellular; FL, full-length; bd, binding domain. ^c^Construct expressed numbered by amino acid position (start – finish / total). ^d^Organism: Mm, Mus musculus; Hs, Homo sapiens; Av, Aequoria Victoria. ^e^Protein family nomenclature according to the Pfam database . ^f^GRAVY, grand average of hydropathicity index. ^g^Highest number of contiguous hydrophobic amino acids (A, V, I, L, W or F). ^h^LC and ^i^CC, number of low complexity and coiled coil regions according to Pfam database.

**Table 2:** N-Terminal fusion expression comparison

	N-TERMINAL FUSION											
	
Protein (domain)	H6		H10		H10-GFP		H10-GST		H10-Trx		H10-MBP	
	
	T	S	T	S	T	S	T	S	T	S	T	S
CASP2	0.00	0.00	0.00	0.00	0.00	0.00	0.00	0.00	0.20	0.20	**2.50**	**2.11**
CCND2	**21.85**	0.00	**12.36**	**5.81**	**6.14**	0.02	1.20	0.00	**12.50**	**4.35**	**8.12**	**3.06**
CD44	0.00	0.00	0.00	0.00	0.00	0.00	nc	nc	0.00	0.00	0.00	0.00
CDK2	**14.81**	1.84	1.03	0.07	nc	nc	**4.88**	**2.17**	2.00	1.54	**25.00**	**25.00**
CDK4	**8.78**	0.71	1.47	1.37	nc	nc	1.32	0.00	nc	nc	**2.79**	0.00
CDKN1B	**7.44**	0.17	0.85	0.31	1.63	0.57	**12.00**	**4.30**	**4.00**	1.69	**8.00**	**5.19**
CDKN2A	0.00	0.00	0.00	0.00	0.00	0.00	0.40	0.32	nc	nc	**3.65**	0.00
Efna1	0.00	0.03	1.50	1.33	**7.47**	0.22	**2.29**	0.06	nc	nc	**5.73**	**3.02**
Efna1 (EC)	**24.74**	0.05	**5.00**	**4.81**	**50.00**	1.71	**28.00**	0.07	**60.00**	**4.10**	**11.93**	**4.93**
Efnb2 (EC1)	**3.58**	0.00	**11.53**	1.18	**10.38**	0.86	**6.43**	1.30	**44.70**	**6.82**	**22.67**	**20.00**
Efnb2 (EC2)	**2.07**	0.04	**3.00**	**2.79**	**50.00**	**6.50**	**55.00**	0.00	nc	nc	**17.00**	**16.00**
EGFR (TK)	0.00	0.00	0.21	0.00	0.00	0.00	0.00	0.00	0.00	0.00	0.00	0.00
Epha2 (LB)	nc	nc	nc	nc	**50.00**	0.43	**2.83**	0.05	**4.92**	1.99	**14.57**	**3.78**
Ephb2 (LB)	**40.00**	0.00	0.66	0.08	**4.93**	0.14	**10.00**	0.03	**29.39**	**2.53**	**20.00**	**2.11**
Ephb2 (SAM)	0.00	0.00	nc	nc	**50.00**	**5.63**	**35.00**	**11.47**	**10.06**	1.34	0.00	0.00
Ephb2 (TK)	0.00	0.00	**65.84**	**15.00**	**20.00**	0.35	**15.00**	0.14	**50.00**	**14.23**	**20.00**	**4.15**
Fli1	**3.82**	0.05	0.89	0.08	2.00	0.00	1.50	0.00	**12.00**	**5.14**	**58.00**	**6.50**
FOS	0.25	0.00	0.00	0.00	0.00	0.00	0.00	0.00	0.49	0.08	0.27	0.00
GATA2	0.33	0.00	0.19	0.07	**2.50**	0.04	0.00	0.00	**5.00**	**3.47**	0.00	0.00
GFP	**60.00**	**60.00**	**25.00**	**25.00**	**25.00**	1.33	**22.08**	**20.00**	**25.00**	**25.00**	**10.00**	**9.83**
GRB2	1.75	0.04	**6.00**	**3.88**	**25.00**	**12.82**	**2.77**	0.84	**13.00**	**11.96**	**18.00**	**16.00**
HRAS	**30.10**	0.34	**6.40**	**5.59**	**6.16**	0.17	**7.37**	0.54	**26.96**	**25.00**	**8.40**	**7.69**
JUN	**40.00**	0.00	**5.84**	1.09	**2.08**	0.00	1.50	0.00	**5.70**	0.41	**30.00**	0.22
MAD	**32.77**	0.15	**3.50**	1.78	**20.66**	0.37	**15.04**	0.13	**9.21**	**4.74**	**4.00**	**4.10**
MAX	nc	nc	**9.43**	1.09	**4.44**	1.18	0.00	0.03	**2.05**	**2.01**	**3.00**	**2.71**
Mdm2	0.00	0.00	1.20	0.91	0.00	0.00	0.00	0.00	**10.00**	**5.57**	**3.60**	**2.65**
Mdm2 (p53-bd)	1.62	0.36	**4.75**	**4.70**	**20.84**	3.20	**20.00**	0.22	**9.54**	**4.72**	**12.00**	**12.00**
MMP1	**2.56**	0.00	0.36	0.10	**11.44**	0.00	**39.63**	0.04	0.32	0.32	**30.00**	0.48
RAF1 (Ras-bd)	**15.48**	**15.00**	**20.00**	**20.00**	**26.92**	0.00	**20.00**	**19.82**	**25.00**	**25.00**	**40.00**	**25.00**
Trp53	0.85	0.00	0.00	0.00	0.00	0.00	0.00	0.00	0.00	0.00	0.00	0.00
AVERAGE	**11.36**	0.81	**7.28**	**3.27**	**10.53**	1.01	**9.02**	1.37	**15.90**	**6.02**	**14.87**	**5.81**

Numbers correspond to total (T) or soluble (S) expression yield (mg/l). Yields greater than 2 mg/l are in bold, nc-not cloned.

**Table 3:** C-Terminal fusion expression comparison

	C-TERMINAL FUSION															
	
Protein (domain)	V5-H6		H10		GFP-H10		GST-H10		Trx-H10		MBP-H10		Dhfr-H10		DHFR-H10	
	
	T	S	T	S	T	S	T	S	T	S	T	S	T	S	T	S
CASP2	0.00	0.00	0.00	0.00	0.00	0.00	0.00	0.00	0.00	0.00	0.00	0.00	0.00	0.00	0.00	0.00
CCND2	0.00	0.00	0.00	0.00	0.00	0.00	0.00	0.00	0.00	0.00	0.00	0.00	0.00	0.00	0.00	0.00
CD44	1.37	0.72	0.00	0.00	0.00	0.00	0.00	0.00	0.00	0.00	0.00	0.00	0.00	0.00	0.00	0.00
CDK2	**23.47**	**12.30**	1.88	1.30	0.70	0.19	0.53	0.08	0.86	0.16	**7.52**	**3.48**	**5.82**	0.51	0.00	0.00
CDK4	0.00	0.00	0.00	0.00	0.00	0.00	0.00	0.00	0.13	0.00	0.00	0.00	**2.07**	0.44	0.00	0.00
CDKN1B	0.00	0.00	0.00	0.00	0.00	0.00	0.00	0.00	0.00	0.00	1.59	0.61	0.00	0.00	**5.76**	0.00
CDKN2A	0.00	0.00	0.00	0.00	0.00	0.00	0.00	0.00	**2.59**	0.56	0.00	0.00	0.48	0.22	0.00	0.00
Efna1	**10.64**	**8.29**	0.00	0.00	0.00	0.00	0.00	0.00	0.24	0.03	**2.30**	1.23	0.00	0.00	**5.24**	0.56
Efna1 (EC)	1.71	0.46	**2.76**	0.58	0.25	0.00	**19.99**	0.24	**3.71**	0.00	**31.94**	**15.60**	**14.01**	0.00	1.10	1.02
Efnb2 (EC1)	**7.91**	0.37	0.43	0.04	0.32	0.04	0.56	0.00	**2.53**	0.00	**6.30**	0.80	0.00	0.24	**5.84**	0.00
Efnb2 (EC2)	**3.57**	0.38	0.52	0.11	0.54	0.06	0.00	0.00	**2.08**	0.00	**3.53**	1.72	**2.59**	0.00	0.00	0.00
EGFR (TK)	1.14	0.00	0.00	0.00	0.00	0.00	0.28	0.00	**12.76**	1.11	**3.03**	0.25	**2.07**	0.00	0.00	0.00
Epha2 (LB)	**3.97**	0.27	0.00	0.00	0.15	0.00	**2.37**	0.00	**4.33**	0.42	**19.33**	**8.60**	**7.42**	0.00	0.00	0.00
Ephb2 (LB)	**15.17**	**7.46**	0.00	0.00	0.00	0.00	0.64	0.00	**2.34**	0.94	**11.93**	**7.93**	**2.48**	0.37	**12.65**	0.00
Ephb2 (SAM)	0.27	0.03	0.00	0.00	0.29	0.08	**43.05**	0.00	0.98	0.10	**156.52**	**20.33**	**33.57**	**7.37**	**2.20**	0.00
Ephb2 (TK)	**43.00**	1.89	**8.85**	0.12	0.00	0.00	**205.64**	**2.13**	**24.04**	0.00	**49.99**	**3.01**	**27.02**	1.06	0.00	0.00
Fli1	0.00	0.00	0.00	0.00	0.00	0.00	0.00	0.00	**3.11**	0.00	1.10	0.00	0.00	0.00	**7.14**	0.00
FOS	**4.72**	0.27	0.22	0.00	0.92	0.49	0.00	0.00	0.11	0.00	1.16	0.62	0.00	0.00	**4.35**	0.00
GATA2	0.00	0.00	0.00	0.00	0.00	0.00	0.00	0.00	0.00	0.00	0.00	0.00	0.00	0.00	0.00	0.00
GFP	**8.14**	1.38	1.11	0.67	**12.94**	**9.86**	**2.03**	**2.01**	**21.50**	**4.78**	**118.38**	**40.52**	**4.66**	**2.93**	**9.56**	**2.74**
GRB2	**15.66**	**5.41**	0.71	0.17	**3.55**	**3.02**	**2.43**	1.70	**5.78**	**3.48**	**110.84**	**14.48**	**4.83**	**3.04**	1.30	0.00
HRAS	**19.21**	**6.44**	0.29	0.25	**11.49**	**6.46**	0.56	0.39	1.06	0.37	**12.32**	**10.07**	**3.21**	**3.01**	0.75	0.57
JUN	**6.75**	0.00	0.31	0.00	0.00	0.00	0.48	0.00	1.41	0.00	**8.81**	0.35	0.00	0.00	**7.79**	0.00
MAD	**9.76**	**2.62**	**3.94**	**5.05**	0.49	0.13	1.37	0.00	**5.91**	**3.34**	**19.02**	**6.94**	1.86	0.13	0.88	0.00
MAX	0.00	0.00	0.00	0.00	0.71	0.53	**101.26**	**80.86**	1.59	0.62	**9.70**	**5.17**	**5.82**	**3.48**	0.94	0.00
Mdm2	0.00	0.00	0.00	0.00	0.00	0.00	0.00	0.00	0.00	0.00	0.00	0.00	0.00	0.00	0.00	0.00
Mdm2 (p53-bd)	1.10	0.25	0.60	0.14	**3.94**	0.33	**2.50**	0.47	**69.23**	**3.03**	**19.57**	**3.82**	**5.46**	0.83	0.00	0.00
MMP1	**12.51**	0.85	0.00	0.00	0.00	0.00	**3.27**	0.00	0.00	0.00	0.00	0.00	**4.29**	0.00	0.52	0.00
RAF1 (Ras-bd)	0.48	0.06	0.00	0.00	0.45	0.16	0.96	0.19	**2.66**	0.71	**5.83**	1.63	**2.08**	1.07	0.00	0.00
Trp53	0.52	0.07	0.00	0.00	0.00	0.00	0.23	0.00	**3.59**	0.91	**5.66**	**2.44**	1.40	0.79	**2.76**	0.00
AVERAGE	**6.37**	1.65	0.72	0.28	1.23	0.71	**12.94**	**2.94**	**5.75**	0.69	**20.21**	**4.99**	**4.37**	0.85	**2.29**	0.16

Numbers correspond to total (T) or soluble (S) expression yield (mg/l). Yields greater than 2 mg/l are in bold.

**Table 4:** Expression levels of mammalian proteins expressed with N-terminal H10-MBP fusions, with selected protein features

No.	Protein	Accession No.	PfamA Domain^a^	Construct	MW (kDa)	hp_aa	LC	Total Expression (mg / l)	Soluble Expression (mg / l)
31	TAL1	P17542	HLH	179–331/331	16.5	3.0	1.0	13.2	8.3
32	ELF1	P32519	Ets	2–619/619	67.4	6.0	5.0	0.0	0.0
33	ELF1	P32519	na^b^	2–167/619	18.0	4.0	2.0	14.6	14.6
34	ELF1	P32519	Ets	204–619/619	45.4	6.0	2.0	0.0	0.0
35	ELF1	P32519	na	316–619/619	32.2	3.0	1.0	0.0	0.0
36	ELF1	P32519	Ets	204–306/619	12.1	6.0	0.0	65.0	19.3
37	Elf1	Q60775	Ets	2–612/612	66.1	6.0	4.0	24.0	23.5
38	Elf1	Q60775	Ets	2–306/612	34.0	6.0	3.0	60.6	27.2
39	Elf1	Q60775	na	2–167/612	17.8	4.0	2.0	8.6	8.4
40	Elf1	Q60775	Ets	204–612/612	44.2	6.0	1.0	15.5	10.4
41	Elf1	Q60775	Ets	204–306/612	12.1	6.0	0.0	18.5	12.1
42	Elf1	Q60775	na	316–612/612	30.7	3.0	0.0	0.0	0.0
43	Gata1	P17679	GATA × 2	2–413/413	42.5	3.0	6.0	16.0	14.3
44	Gata1	P17679	GATA × 2	2–319/413	33.8	3.0	5.0	67.0	15.8
45	Gata1	P17679	na	2–182/413	18.6	3.0	3.0	9.7	7.2
46	Gata1	P17679	GATA × 2	191–413/413	23.1	3.0	5.0	19.7	9.6
47	Gata1	P17679	GATA × 2	191–319/413	14.3	3.0	0.0	0.0	0.0
48	Gata2	O09100	GATA × 2	2–480/480	50.3	5.0	5.0	0.0	0.0
49	Gata2	O09100	na	2–189/480	19.4	4.0	2.0	0.0	0.0
50	Gata2	O09100	GATA × 2	275–480/480	22.4	5.0	2.0	0.0	0.0
51	Gata2	O09100	GATA × 2	275–402/480	14.2	1.0	1.0	0.0	0.0
52	Fli1	P26323	SAM_PNT, Ets	2–452/452	50.9	3.0	1.0	0.0	0.0
53	Fli1	P26323	SAM_PNT, Ets	2–363/452	41.7	3.0	0.0	134.5	61.0
54	Fli1	P26323	SAM_PNT	2–198/452	22.2	3.0	0.0	121.2	86.8
55	Fli1	P26323	SAM_PNT, Ets	114–452/452	38.6	3.0	1.0	61.0	38.2
56	Fli1	P26323	SAM+ETS	114–363/452	29.4	3.0	0.0	96.5	73.1
57	Fli1	P26323	SAM_PNT	114–196/452	10.0	3.0	0.0	71.8	51.7
58	Fli1	P26323	Ets	280–452/452	20.1	3.0	1.0	28.6	16.3
59	Fli1	P26323	Ets	280–363/452	10.9	3.0	0.0	23.4	23.0
60	Lmo2	P25801	LIM × 2	2–158/158	18.2	3.0	0.0	106.7	23.8
61	Ldb1	P70662	LIM_bind	2–375/375	42.6	3.0	2.0	0.0	0.0
62	Ldb1	P70662	LIM_bind	2–273/375	31.9	3.0	0.0	133.8	62.0
63	Ldb1	P70662	LIM_bind	275–375/375	10.5	2.0	1.0	2.0	1.7
64	Lyl1	P27792	HLH	40–278/278	26.2	3.0	0.0	3.8	2.6
65	Lyl1	P27792	HLH	40–215/278	19.6	3.0	0.0	4.2	2.5
66	Lyl1	P27792	na	40–135/278	10.3	3.0	0.0	3.6	1.7
67	Lyl1	P27792	HLH	150–278/278	14.8	3.0	0.0	40.1	32.2
68	Lyl1	P27792	HLH	150–215/278	8.2	3.0	0.0	60.3	20.8
69	Ttr	P07309	transthyretin	20–147/147	13.6	5.0	0.0	59.2	49.7
70	Pin1	Q9QUR7	WW, Rotamase	2–163/163	18.2	2.0	0.0	36.9	19.4
71	Whsc1	Q7TSF5	PHD × 2, PWWP, SET	2–558/558	63.8	4.0	2.0	9.4	1.3
72	Whsc1	Q7TSF5	PHD, PWWP, SET	2–373/558	43.0	4.0	0.0	21.6	12.4
73	Whsc1	Q7TSF5	PHD, PWWP	2–149/558	17.2	4.0	0.0	0.0	0.0
74	Whsc1	Q7TSF5	PWWP, SET, PHD	70–558/558	56.1	4.0	2.0	5.1	2.1
75	Whsc1	Q7TSF5	PWWP, SET	70–373/558	35.3	4.0	0.0	18.2	17.9
76	Whsc1	Q7TSF5	PWWP	70–149/558	9.5	4.0	0.0	56.8	14.6
77	Whsc1	Q7TSF5	SET	249–373/558	14.3	4.0	0.0	34.7	22.6
78	Maat1	NM_024227	na	2–257/257	30.0	3.0	0.0	2.7	2.4
79	BC031407	NM_145596	na	2–630/630	67.3	6.0	6.0	0.0	0.0
80	BC031407	NM_145596	na	2–455/630	48.5	6.0	5.0	0.0	0.0
81	BC031407	NM_145596	na	2–179/630	19.4	4.0	1.0	9.6	8.7
82	BC031407	NM_145596	na	178–630/630	48.0	6.0	5.0	0.0	0.0
83	BC031407	NM_145596	na	178–455/630	29.1	6.0	4.0	0.0	0.0
84	BC031407	NM_145596	na	413–630/630	23.7	4.0	1.0	0.0	0.0
85	Bzrp2	P50637	TspO_MBR	2–169/169	18.7	4.0	0.0	0.0	0.0
86	MGC19339	NM_145954	Aldedh	40–486/803	47.0	5.0	2.0	37.2	18.7
87	Bsg	NM_009768	Ig × 2, V-set	28–323/389	32.4	4.0	0.0	61.2	36.6
88	Snx15	NM_026912	PX, MIT	2–337/337	37.6	4.0	1.0	32.4	32.0
89	Snx15	NM_026912	PX	2–226/337	25.6	4.0	1.0	20.0	18.8
90	Atp2b2	Q9R0K7	Cation_ATPase_N	2–94/1198	10.4	2.0	0.0	42.1	24.7
91	Atp2b2	Q9R0K7	Cation_ATPase_N	1039–1198/1198	17.9	2.0	3.0	6.1	5.0
92	cdh23	Q99PF4	Cadherin	33–132/3354	11.1	4.0	0.0	174.7	38.8
93	Myo15	Q9QZZ4	SH3_2	2847–2937/3511	9.8	4.0	0.0	8.6	0.0
94	Myo7a	P97479	SH3_1	1602–1672/2215	7.8	4.0	0.0	76.0	35.4
95	tmc1	Q8R4P5	na	2–193/757	22.9	3.0	3.0	0.0	0.0
96	Trvp4	NM_022017	na	500–718/871	24.6	9.0	0.0	0.0	0.0
97	Whrn	XM_196324	PDZ	811–908/908	11.0	3.0	0.0	34.1	31.1
98	Espn	NM_019585	WH2	2–253/253	28.0	3.0	2.0	5.5	3.8
99	Map2	P20357	Tubulin-binding	1657–1755/1828	10.6	2.0	0.0	47.1	46.8
100	Prom	O54990	Prominin	124–162/867	4.3	2.0	1.0	16.3	8.5
101	GluR1	P23818	ANF_receptor	19–538/907	59.0	4.0	0.0	0.0	0.0
102	GluR2	P23819	ANF_receptor	22–545/883	58.6	4.0	0.0	0.0	0.0
103	Grin1	P35438	na	834–938/938	12.0	5.0	0.0	13.8	13.8
104	Grin2a	P35436	na	23–555/1464	59.9	6.0	0.0	58.5	8.2
105	Grin2b	Q01097	Lig_chan	656–817/1482	18.1	4.0	0.0	0.0	0.0
106	Dlgh2	NM_011807	PDZ	419–530/852	11.7	4.0	0.0	13.8	13.7
107	Dlgh4	Q62108	PDZ	311–394/724	8.7	4.0	0.0	37.4	29.2
108	Dlgh3	P70175	PDZ	402–509/849	11.7	5.0	0.0	0.0	0.0
109	Dlgh1	U93309	PDZ	432–572/927	15.1	5.0	0.0	26.7	23.4
110	Syngap1	XM_139847	RasGAP	405–615/1318	23.9	3.0	0.0	0.0	0.0
111	Grip1	Q925T5	PDZ	1–112/1034	9.6	3.0	0.0	0.0	0.0
112	Homer1	Q9Z2Y3	WH1	2–107/354	12.1	3.0	0.0	17.9	17.6
113	Homer3	Q99JP6	WH1	2–110/356	39.3	4.0	0.0	0.0	0.0
114	TtyhI	Q9EQN7	na	263–450/450	20.6	5.0	0.0	35.6	28.3
115	1500001H12RIKEXT2	NM_021316	na	2–149/149	14.8	5.0	3.0	66.1	66.1
116	Ext2	NM_010163	na	99–392/718	33.0	4.0	0.0	18.5	3.5
117	KIAA1136	Q9ULT3	na	45–214/597	19.2	2.0	0.0	26.8	10.4
118	G2	Q12914	na	1046–1692/1692	71.3	5.0	3.0	0.0	0.0
119	KIAA1549	Q9HCM3	na	184–464/1865	29.5	5.0	4.0	3.5	0.0
120	Nfkb1	P25799	RHD	39–365/971	36.7	5.0	0.0	27.1	22.7
121	Nfkb1	P25799	RHD, TIG, Ank × 6, Death	2–971/971	105.5	7.0	3.0	0.0	0.0
122	RelA-p65	Q04207	RHD, TIG	18–306/549	32.9	4.0	0.0	24.1	18.2
123	RelA-p65	Q04207	RHD, TIG	2–549/549	60.0	5.0	2.0	0.0	0.0
124	RelB	Q04863	RHD, TIG	102–418/558	35.8	4.0	0.0	46.0	25.9
125	myog	P12979	HLH, Basic	2–224/224	25.1	3.0	1.0	25.8	12.4

Features listed as Table 1 except: ^a^PfamA domains contained within expressed protein and ^b^na – no PfamA domains annotated.
